# Genetic insights into the peoples who shaped the American continent

**DOI:** 10.1590/1678-4685-GMB-2025-0244

**Published:** 2026-04-03

**Authors:** Gustavo Medina Tavares, Bruna Oliveira Missaggia, Thaynara Lima, Mariana Marcano-Ruiz, Maria Thereza Schmitt Mesquita, Guilherme Baldo, Márcio Dorn, Tábita Hünemeier, Maria Cátira Bortolini

**Affiliations:** 1 Universidade Federal do Rio Grande do Sul, Instituto de Biociências, Departamento de Genética, Programa de Pós-Graduação em Genética e Biologia Molecular (PPGBM), Laboratório de Evolução Humana e Molecular (LEHM), Porto Alegre, RS, Brazil.; 2 Hospital de Clínicas de Porto Alegre, Centro de Pesquisa Experimental, Laboratório Basic Research and Advanced Investigation in Neuroscience (BRAIN), Porto Alegre, RS, Brazil.; 3 Hospital de Clínicas de Porto Alegre, Centro de Pesquisa Experimental, Laboratório de Células, Tecidos e Genes (CTG), Porto Alegre, RS, Brazil.; 4 Universidade Federal do Rio Grande do Sul, Centro de Biotecnologia (CBiot), Instituto de Informática (INF), Departamento de Informática Teórica, Structural Bioinformatics and Computational Biology Lab, Porto Alegre, RS, Brazil.; 5 Sede da Universidade Federal do Rio Grande do Sul, Instituto Nacional de Ancestralidade Genômica Brasileira (AncesGen), Porto Alegre, RS, Brazil.; 6 Universidade de São Paulo, Departamento de Genética e Biologia Evolutiva, Instituto de Biociências, São Paulo, SP, Brazil.; 7 Universitat Pompeu Fabra, Departament de Medicina i Ciències de la Vida, Institut de Biologia Evolutiva, Barcelona, Spain.

**Keywords:** Native American, Indigenous, admixture, genome editing, adaptation

## Abstract

The initial peopling of America left a deep genetic legacy in Indigenous peoples and their admixed descendants. This narrative review recenters studies involving Indigenous populations and, inspired by the work of Francisco M. Salzano and Darcy Ribeiro’s historical and cultural framework, adopts the working notions of “witness,” “introduced,” “transplanted,” and “new” genetic signatures. We first clarify terminology to avoid neocolonial bias, using America to denote the continent and Native American to refer to all Indigenous peoples of America, and then synthesize the literature on initial peopling, post-contact demography, and natural selection, with particular emphasis on Brazil. We also present an illustrative example drawn from ongoing research conducted by our group, using genome editing to investigate a candidate adaptive allele in the context of high-altitude adaptation. Finally, we connect evolutionary history to contemporary health, highlighting mitonuclear interactions, dietary transitions, and pathogen exposures that may modulate disease risk, with implications for precision public health. Collectively, this review showcases ancestry-aware approaches tailored to Native American contexts and demonstrates why models developed elsewhere should not be uncritically extrapolated to America, advancing a continent-wide, Brazil-anchored perspective on Indigenous resilience and scientific significance.

## Introduction

Uncovering the history of Native Americans and their admixed descendants reveals the legacy of one of *Homo sapiens’* most consequential dispersals: the successful peopling of America. Beyond the initial migrations, today’s genetic landscape reflects a complex history shaped by successive layers of admixture, first among Indigenous populations and, after contact, between these groups and individuals of African and European ancestry. This history illustrates *H. sapiens* mobility, evolutionary dynamics, and our capacity to adapt to diverse and often extreme environments. Studying Native American populations is therefore not only scientifically valuable but also essential for a comprehensive understanding of human biology, particularly in light of the gene-culture coevolution that has shaped our species’ diversity and survival.

Despite their importance, Native American peoples remain underrepresented in genetic research compared to Europeans and their descendants. Most genomic studies remain Eurocentric, with research frameworks and clinical applications primarily based on individuals of European ancestry (Sirugo et al., 2019; Fatumo et al., 2022; Marcano-Ruiz et al., 2023). This imbalance also affects admixed populations across America, despite the efforts of pioneer researchers, such as Professor Francisco M. Salzano (1928-2018), and stands in stark contrast to the scientific value of the research he helped inaugurate, whose legacy continues to inspire ongoing work.

Expanding research initiatives to include Indigenous and other historically underrepresented populations is essential to capture human genetic diversity and population-specific characteristics. Incorporating high-quality genetic data, advanced bioinformatics, and functional studies fosters a more equitable and robust understanding of human variation, its causes and consequences, and supports the development of precision medicine and context-appropriate health policies. These efforts also align with the United Nations’ Sustainable Development Goals, particularly Goals 3 (Good Health and Well-being) and 10 (Reduced Inequalities), while reframing narratives about Native Americans beyond colonization to emphasize their genetic diversity, deep historical roots, and contributions to reconstructing human population history, adaptation, resilience, and resistance.

Professor Francisco M. Salzano and collaborators began with classical genetic markers, especially blood groups, across diverse Native American populations (Salzano, 1957). Studies with the Kaingang people integrated demographic, morphological, and serological data (Salzano, 1961a, b, c) and yielded landmark papers in *Science* (absence of abnormal hemoglobin variants; Tondo and Salzano, 1960) and *Nature* (natural selection intensity; Salzano, 1963). Subsequent work broadened to other Indigenous peoples while consistently integrating genetic, demographic, and anthropological approaches. This body of research, built with a wide network of collaborators and students, was synthesized in Salzano and Callegari-Jacques (1988) and in Salzano (2019), his final book, published posthumously. The Indigenous genetic contribution within admixed populations, including to his own people, the *Gaúchos*, is documented in Salzano and Bortolini (2002), Marrero et al. (2007), and Salzano and Sans (2014).

Equally significant was Salzano’s role in establishing ethical standards for research involving Indigenous populations. As noted by Bortolini (2019), his contributions to the field of bioethics began at a time when the term itself was scarcely known. In the 1960s, he was among a select group of experts invited by the World Health Organization (WHO) to help define ethical principles for genetic and evolutionary studies involving human participants (WHO, 1964, 1968). These early efforts were particularly relevant to research involving Native American populations, emphasizing the importance of informed consent, which at the time was obtained through oral agreements, always with the support of state institutions such as Brazil’s National Foundation of Indigenous Peoples (FUNAI). They also underscored the importance of respecting participant privacy and well-being, ensuring equitable access to healthcare, maintaining transparency regarding research outcomes, and safeguarding cultural integrity (WHO, 1964, 1968). Salzano remained committed to addressing ethical challenges throughout his career, including the emergence of anti-scientific discourse and strategies to confront it, as reflected in some publications (*e.g*., Salzano, 2015). His ethical engagement was inseparable from his scientific agenda and remains a cornerstone for those continuing this line of research. More recently, Dent (2024), while acknowledging the historical relevance of earlier practices and Professor Salzano’s role, emphasizes that contemporary approaches call for ongoing consent and active Indigenous participation throughout the research process.

Here, we present a non-systematic narrative review, a qualitative synthesis of selected literature that offers context and interpretation rather than exhaustive coverage (Green et al., 2006; Ferrari, 2015; Sukhera, 2022). We highlight findings that have shaped current knowledge on Native American and admixed populations, reflecting both continuity and innovation within the long-standing research tradition initiated by Professor Salzano, to which we belong and to which we actively contribute to honoring his legacy. Specifically, we discuss appropriate terminology, the peopling of America, the dynamics of population admixture that shaped genetic diversity across the continent; examine adaptations that arose during the dispersal of Native Americans, emphasizing evolutionary pressures with biomedical relevance; transpose Darcy Ribeiro’s categories (Ribeiro, 1970a, b) into a genetic framework of “witness,” “introduced,” “transplanted,” and “new” genetic signatures; and explore studies on Indigenous health that show how genomic insights can inform public health strategies and precision medicine. We also present thought-provoking new results illustrating how emerging methodologies can deepen our understanding of these populations.

## The first inhabitants and the onset of European presence in America

### Naming, perceptions, and terminological shifts

In 1492, the navigator Christopher Columbus and his crew believed they had reached the Indies, hoping to encounter the wonders described in Marco Polo’s accounts. Rather than the imagined Asian civilizations, they encountered a land already home to numerous people, with complex societies and cities as large as or larger than Europe’s biggest centers of the time (Salzano and Bortolini, 2002). From this misperception arose the designation “Indians” for the local populations. A few years later, while mapping the Brazilian coastline in the service of Portugal, Amerigo Vespucci adopted a different perspective from Columbus. He recognized that the newly encountered territories formed an extensive new continent, the “New World”. In recognition of his contribution, the continent was eventually named *America* after him (Salzano and Bortolini, 2002).

Over the centuries, the label “Indian” became increasingly associated with stereotypes and colonial bias. Alternative designations such as *Indigenous* and *Native American* were gradually introduced to foster more accurate and respectful representation. It is also important to emphasize that the term *America* properly refers to the entire continent, even though it is often used as a synonym for the United States of America. Tsosie et al. (2020), in a seminal article authored by Indigenous scholars from the U.S., proposed that the term *Native American* be used specifically within the U.S. political context. While this perspective is understandable within that framework, restricting both *America* and *Native American* to U.S.-centered categories risks reinforcing limited and exclusionary interpretations. Accordingly, throughout this review we adopt a broader, continental usage of these terms, aiming to promote a more inclusive and representative view of all peoples of America.

### The “discovery” and the true first inhabitants

**Figure 1:**
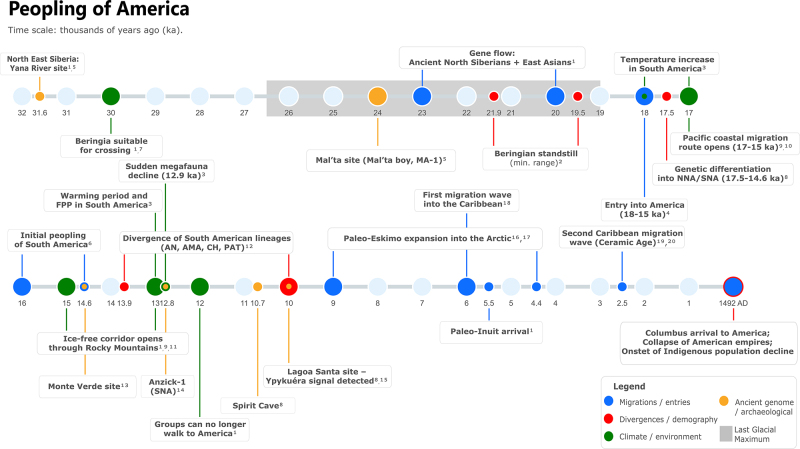
Timeline of selected major events in the peopling of America. Circles indicate reference dates (ka, thousands of years before present) along a horizontal axis from past to present; colors denote event categories (e.g., migrations, climate/environment). Some ranges are shown by their upper bound. Abbreviations: NNA (Northern Native American), SNA (Southern Native American), FFP (Fishtail projectile points), AN (Andes), AMA (Amazon), CH (Chaco), PAT (Patagonia). References: (1) (Willerslev and Meltzer, 2021); (2) (Pinotti et al., 2019); (3) (Prates and Perez, 2021); (4) (Gómez-Carballa et al., 2018); (5) (Raghavan et al., 2014a); (6) (Brandini et al., 2018); (7) (Niedbalski and Long, 2022); (8) (Moreno-Mayar et al., 2018); (9) (Perego et al., 2009); (10) (Lesnek et al., 2018); (11) (Potter et al., 2018); (12) (Gusareva et al., 2025); (13) (Dillehay, 2009; Dillehay et al., 2015); (14) (Rasmussen et al., 2010); (15) (Skoglund et al., 2015; Castro e Silva et al., 2021); (16) (Harritt, 1998); (17) (González-José et al., 2008; Bortolini et al., 2014); (18) (Nägele et al., 2020); (19) (Fernandes et al., 2021); (20) (Forbes-Pateman et al., 2022).

The arrival of Europeans in America has often been described as a “discovery,” a term rooted in a strictly Eurocentric perspective. In reality, the continent was already inhabited by millions of Indigenous peoples, and the story of the first humans who arrived and colonized America is both remarkable and interwoven with drama, a narrative that has sparked curiosity, scientific and beyond, for centuries (Salzano and Bortolini, 2002; Santos et al., 2007). Genetics has become a valuable tool for uncovering past evolutionary and demographic events in the peopling of the American continent. Some important aspects of this process are briefly introduced in this section and summarized in Figure 1. For a more detailed and recent discussion, see Hünemeier et al. (2025), and for a more pluralistic perspective, see Bisso-Machado and Fagundes (2019).

In brief, the settlement of America is now recognized as a Late Pleistocene migration resulting from admixture between Ancient North Siberians (*e.g*., the Mal’ta individual, ~24 kya) and East Asians (Raghavan et al., 2014a). These people reached Beringia, where isolation during the Last Glacial Maximum (~26.5-19 kya) led to genetic divergence over 2.4-9 thousand years (Bonatto and Salzano, 1997; Tamm et al., 2007; Fagundes et al., 2008; Llamas et al., 2016; Pinotti et al., 2019), giving rise to distinct Native American lineages that split into northern (NNA) and southern (SNA) Native American branches around 17.5-14.6 kya (Moreno-Mayar et al., 2018).

Genetic data also indicate a small founding population, with autosomal and mtDNA estimates ranging from a few dozen to a few thousand individuals (Fagundes et al., 2007, 2008; Kitchen et al., 2008; Ray et al., 2010). Early Y-chromosome studies (Pena et al., 1995; Bortolini et al., 2003) and high-resolution sequencing (Pinotti et al., 2019) further support few Siberian paternal founders and the origin of a small set of exclusive Y haplogroups (*e.g*., Q-M3 in Beringia), consistent with a severe bottleneck. A rapid Pacific coastal dispersal was also detected with Y dataset (Bortolini *et al*., 2003), consistent with archaeological evidence supporting a swift Paleo-American expansion along the Pacific coast, reaching southern Chile by approximately 14.5 thousand years ago (Dillehay, 2009; Dillehay et al., 2015).

Beyond the canonical Siberian ancestry, our group, in collaboration with an international research team, detected a “ghost” population signal, named *Ypykuéra* (“Population Y”), inferred from the surprising affinity between some Native Amazon groups and modern Australo-Melanesians (Skoglund et al., 2015). Importantly, this signal does not correspond to canonical East Asian or Siberian ancestry but rather reflects a previously unrecognized non-Siberian/non-East Asian ancestral component, which was subsequently found outside the Amazon region and shown to be widespread across South America (Castro e Silva et al., 2021). It was detected in present-day Indigenous groups from the Amazon, the Central Plateau, and the Pacific coast, as well as in ancient individuals from the Brazilian Atlantic coast and the Central Plateau. This signal compels a revised framework for the peopling of America that accommodates non-Siberian/non-East Asian sources of ancestry in Beringia, likely involving contributions from South Asia.

Subsequent northern movements (Paleo-, “Neo”-Eskimo/Inuit, and Na-Dene) further shaped regional patterns (Harritt, 1998; Reich et al., 2012; Raghavan et al., 2015). For instance, the Paleo-Eskimo (*e.g*., Saqqaq, Dorset) expansion during the mid-Holocene (~5.5-4.0 kya) is associated with the spread of the Arctic Small Tool tradition (Harritt, 1998; Raghavan *et al*., 2014b). Craniofacial evidence indicates that more derived extreme East Asian-like morphological traits emerged earlier in the middle Holocene (~7-7.5 kya) and reached America at some point thereafter, potentially as part of broader post-Beringian population dynamics that included the earliest Paleo-Eskimo movements, or alternatively through later low-level but continuous circumarctic interactions between East Asia and America (González-José et al., 2008; Bortolini et al., 2014). This was followed by the Thule migration (~0.7 kya), which largely replaced Paleo-Inuit populations while retaining some degree of gene flow (Raghavan *et al*., 2014b), giving rise to what is archaeologically recognized as the Neo-Eskimo tradition (Friesen, 2004). An additional pulse contributing to Na-Dene groups was proposed (Reich *et al*., 2012); however, subsequent genomic analyses indicate that Na-Dene populations share a primary ancestry with other Native Americans, with their distinctiveness better explained by limited subsequent admixture rather than by a fully independent migration (Raghavan *et al*., 2015).

Native American groups gradually dispersed across the continent, leading to regional population growth, differentiation, admixture, and local adaptation. These processes generated remarkable biological and cultural diversity, most pronounced in the pre-contact period and still observable today. Large empires flourished in Mesoamerica and the Andes, producing complex urban centers, some larger than important European cities (Salzano and Bortolini, 2002), while others remained isolated, maintaining hunter-gatherer traditions (currently about 114 isolated forest peoples are registered in the Brazilian Amazon; FUNAI, 2021). These contrasting histories have shaped distinct genetic patterns, *e.g.*, Andean populations show lower inter-population differentiation than lowland Amazonians (Tarazona-Santos et al., 2001). This diverse demographic landscape was profoundly altered after 1492, when European colonization brought extensive admixture, epidemics, and sociopolitical disruption, transforming Native American population dynamics across the continent.

### Admixed America

Following European arrival, America underwent rapid population diversification. Early entrants were few relative to the mass European migrations of the late 19th-early 20th centuries. Most migrants settled in the United States, followed by Argentina, Canada, and Brazil; Cuba and Uruguay received smaller shares, and other countries much smaller contingents (Nugent, 1992; Sánchez-Alonso, 2019). During the transatlantic slave trade, over ten million Africans were forcibly brought to America, chiefly to the Caribbean and Brazil (Curtin, 1972; Fortes-Lima and Verdu, 2021). Additional, smaller inflows included East Asian migrants, mainly Chinese and Japanese, and Arabic-speaking migrants from Ottoman-ruled regions (see Figure S1 and the references therein).

*Admixed Brazil*

The Brazilian population represents a recent and extraordinary example of encounters among culturally and biologically diverse groups of *H*. *sapiens*, forged through successive layers of colonization, admixture, and immigration. At the time of European arrival, about 5-10 million Native Americans inhabited the territory, speaking more than 1,000 languages (Rodrigues, 2005; Vainfas, 2007; Castro e Silva et al., 2022). Over the following centuries, approximately 5 million Europeans migrated to the region (Salzano and Bortolini, 2002; Nunes et al., 2025), and about 5 million enslaved Africans were brought through the transatlantic slave trade (Salzano and Bortolini, 2002). Additional migrations from Europe, East Asia, and the Levant region during the 19th and 20th centuries further enriched this demographic mosaic (Salzano and Bortolini, 2002).

It is important to note that the above account of admixture is a simplification: all contributing groups were themselves heterogeneous and arrived at different times over several centuries. The Portuguese, for example, had been shaped by successive migrations and long-standing interconnections among diverse peoples. Medieval Islamic rule in Iberia (*al-Andalus*, the territories under Muslim rule), often described as the Moorish domain, added a documented wave of trans-Gibraltar mobility (soldiers, settlers, merchants, and enslaved people) that intensified exchanges between North Africa and Iberia. Genetic signatures indicate that this episode layered additional North African ancestry onto much older, bidirectional flows, making the Moorish period one chapter in a deeper, continuous history of western Mediterranean connectivity (Gonçalves et al., 2005; Marques et al., 2015; Hernández et al., 2020; Roca-Rada et al., 2025). Africans brought to Brazil originated from diverse West and Central African regions (Bortolini et al., 2004; Silva et al., 2006; Hünemeier et al., 2007), whereas Indigenous peoples contributed with marked local differences in cultural and genetic diversity across Brazil’s vast territory (Salzano and Callegari-Jacques, 1988; Salzano and Bortolini, 2002; Marrero et al., 2007). Furthermore, early admixture in Brazil was strongly gender-asymmetric, primarily involving Portuguese men, Indigenous women, and later African women, forming the basis of colonial society, a pattern clearly revealed by mtDNA and Y-chromosome studies (Alves-Silva et al., 2000; Carvalho-Silva et al., 2001; Salzano and Bortolini, 2002; Nunes et al., 2025).

The amplitude of this demographic complexity is now revealed through genomic data, allowing fine-scale reconstruction of the underlying historical processes. The most comprehensive genomic analysis of Brazilians to date was recently published, including overall coordination and contributions from members of our team. Nunes et al. (2025) provide the most complete and robust estimates of the genetic composition of present-day Brazilian populations across all major regions. Using 2,723 high-coverage whole genomes, an Indigenous autosomal ancestry of 13.4% was estimated, higher than earlier genome-wide estimates (7-9%; Kehdy et al., 2015; Ruiz-Linares et al., 2014). Notably, the proportion of Native American ancestry varies across Brazilian regions: Indigenous ancestry reaches its highest levels in the North (~30%), African ancestry is enriched in the Northeast (~50%), and European ancestry predominates in the South (~70-75%), while the Southeast shows intermediate values (~65-70% European, ~20-25% African, and ~10% Indigenous). Moreover, comparative analyses indicate that Northern Brazilians share closer genetic affinities with Amazonian Indigenous groups, Northeasterners with West and Central African populations, and Southern Brazilians with Iberian and Central European groups, reflecting the heterogeneous demographic histories that shaped each region (Nunes *et al*., 2025).

As already noted, a substantial portion of Brazilian Native American ancestry is maternally inherited, with mtDNA analyses indicating Indigenous contributions of approximately 34.8%, compared to only 2.4% based on Y-chromosome data. African ancestry accounted for 42.5% of mtDNA lineages and 25.5% of Y-chromosome lineages, whereas European ancestry represented 21.9% of mtDNA and 71.1% of Y-chromosome lineages (Nunes et al., 2025), corroborating a marked sex-biased admixture pattern in Brazil. This notable contribution of Native American women to the Brazilian gene pool contrasts with the minimal genetic contribution of Indigenous men relative to that of European men, highlighting a brutal, though not unique, facet that accompanied the process of territorial conquest. Notable differences were also observed across states and regions (Nunes *et al*., 2025). This initial pattern of sex-biased mating in the early admixture events, evidenced by uniparental markers, is replaced by assortative mating in more recent generations, indicating that marriages tend to occur preferentially between individuals with similar ancestral or admixture profiles. In addition, Nunes *et al*. (2025) identified local ancestry at the individual genomic level, providing an unprecedented context for understanding the profile and dynamics of admixture and its consequences, including its relevance for adaptation on the continent and for differential susceptibility to diseases of modernity, as explored in the following sections.

## Witness, transplanted, and new genetic ancestries: Brazil as a case study

In “*The Culture-Historical Configurations of the American Peoples*” (Ribeiro, 1970a) and “*As Américas e a Civilização*” (Ribeiro, 1970b), the Brazilian anthropologist Darcy Ribeiro (1922-1997) proposed a historical-sociocultural framework to interpret population formation in America. Within this framework, he outlined four broad categories, three of which are pertinent here. First, “witness peoples” (*povos-testemunho*) are groups that had developed complex civilizations before European colonization, such as Mesoamerican and Andean societies, which persisted even after the fall of the Aztec, Maya, and Inca empires and the colonization process (*e.g*., Mexico and Peru). Second, “new peoples” (*povos novos*) emerged from extensive admixture between colonizers (Europeans) and the colonized (Native Americans and Africans), yielding new cultural and demographic configurations, as in Brazil. Third, “transplanted peoples” (*povos transplantados*) refers to settler communities primarily composed of Europeans who migrated with families, traditions, and economic practices, largely preserving their original ethnic structures with only minor or superficial modifications (*e.g*., United States and Canada).

We transpose Ribeiro’s historical and cultural categories to ancestry patterns for analytical purposes, using “genetic signature” as a general descriptor of inherited variation. This transposition is not a literal application of Ribeiro’s framework; rather, it adapts his conceptual distinctions to the genomic level, where the units of analysis differ fundamentally from the sociocultural entities he originally described. By genetic signature, we refer broadly to genomic segments that vary in scale, ranging from single variants to multi-SNP haplotypes and extended chromosomal tracts on the autosomes or sex chromosomes, as well as mitochondrial genomes. Such signatures may be characteristic of particular populations either by exclusivity or by substantially higher frequency relative to other continental groups. 

We use the term “witness genetic signatures” to describe genetic patterns of Native American origin that trace back to the populations present in the continent before European expansion. Many of these signatures persist in present-day Indigenous groups and in admixed populations, sometimes acting as reservoirs even when uncommon among contemporary Indigenous communities. The term “witness” is used metaphorically to emphasize their evidentiary value for reconstructing demographic and evolutionary histories in contexts where written records are sparse or absent.

To adapt Ribeiro’s framework to genomic data, we distinguish “transplanted” from “introduced genetic signatures”. We refer to “transplanted genetic signatures” as those brought by migrant groups whose genetic profiles remained relatively preserved because they formed comparatively endogamous communities upon arrival in Brazil. This category includes, for example, European (*e.g*., German, Italian, Polish, etc.), Japanese and Middle Eastern immigrants from the nineteenth and twentieth centuries, whose genetic contributions did not substantially enter the early admixture processes that shaped most Brazilian genomes.

By contrast, we create the category of “introduced genetic signatures” to describe ancestry components incorporated through gene flow during the formative period of widespread admixture in Brazil. This category includes European components associated with early colonial mixing and African components forcibly brought through the transatlantic slave trade. These signatures became integrated into local populations primarily through extensive admixture rather than through the establishment of cohesive migrant communities.

Historical recombination between the “witness and introduced” European and African signatures, and, more rarely, with “transplanted signatures”, has generated the mosaic genomes observed in contemporary Brazilian populations. We refer to these historically contingent combinations as “new genetic signatures”. These labels describe genomic architecture only; they do not define cultural belonging, identity, or social membership and do not supersede self-identification or community-defined categories.

**Figure 2:**
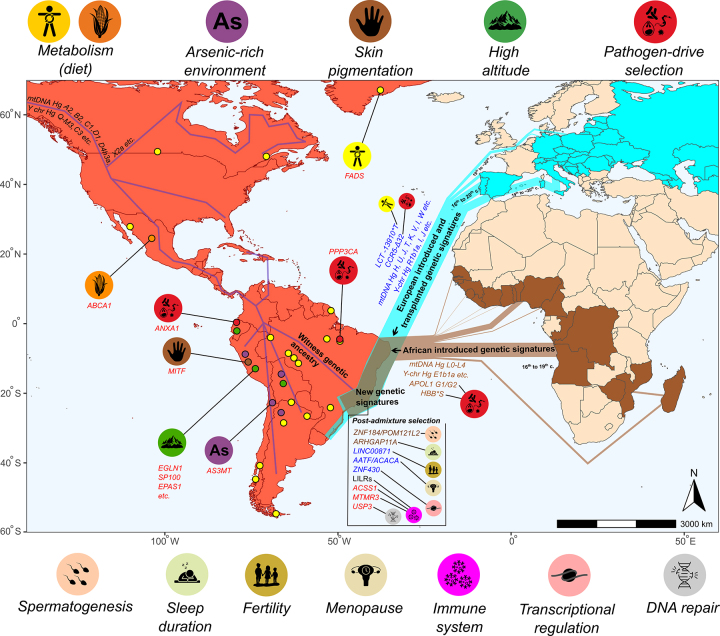
Distribution of genes or genomic regions across the American continent, with a focus on Brazil, illustrating different categories of genetic signatures. These include “witness genetic signatures” (Native American-derived gene variants and mtDNA/Y-chromosome lineages), “introduced genetic signatures” (European and African gene variants and mtDNA/Y-chromosome lineages incorporated into local populations through historical admixture, including the transatlantic slave trade), “transplanted genetic signatures” (gene variants and mtDNA/Y-chromosome lineages retained within relatively endogamous migrant communities of mainly European origin), and “new genetic signatures” (mosaic genomic patterns resulting from recombination among “witness”, “introduced”, and, more episodically, “transplanted signatures”). The variants depicted are discussed throughout the main text in the context of admixture and adaptation, including signatures of natural selection acting either on the continent of origin or after admixture in the Americas. In the figure, red denotes Native American genetic signatures, blue denotes European genetic signatures, and brown denotes African genetic signatures.

In the uniparental context, *i.e.*, mtDNA and the non-recombining portion of the Y chromosome, our framework maps cleanly onto Brazil’s history: “witness genetic signatures” include Indigenous mtDNA (the major A-D haplogroups; Alves-Silva et al., 2000) and Y-chromosome major haplogroups Q-M3 and C (Pinotti et al., 2019; Resque et al., 2016); “Introduced genetic signatures” comprise European mtDNA lineages (*e*.*g*., H, U, J, T; Alves-Silva *et al*., 2000) and Y-chromosome haplogroups (*e.g*., R1b, I, J; Resque *et al*., 2016), as well as African mtDNA L clades (*e*.*g*., L0-L4; Alves-Silva *et al*., 2000; Hünemeier et al., 2007) and Y-chromosome lineages such as E1b1a, which were forcibly incorporated through the transatlantic slave trade (Figure 2). In admixed Brazilians, these genetic elements show a marked sex-biased pattern, European Y chromosomes alongside Native or African mtDNA, as has been demonstrated (Salzano and Bortolini, 2002) and further refined with genomic data (Nunes et al., 2025).

A recent example is our study of 467 urban, admixed Brazilian COVID-19 patients (Tavares et al., 2025), which tested whether mitochondrial genetic ancestry and ancestry‐defining mtDNA coding variants relate to clinical outcomes. Using classical statistical tests and interpretable machine‐learning models on protein‐coding mtDNA variants, we found that the Native American-specific haplogroup A2, particularly its defining nonsynonymous substitutions, with the clearest signal in cytochrome c oxidase subunit II (*MT-CO2*), was associated with the death outcome, indicating that carriers among Native Americans and their descendants may face increased COVID-19 mortality risk. Although single-variant effects were modest, the aggregate signal is consistent with a multifactorial disease architecture and suggests that mtDNA lineage distributions have been shaped by selection imposed by infectious pressures. Accordingly, A2, a “witness genetic signatures” is likely nonneutral, reflecting a long-standing balance between mitochondrial bioenergetics and antiviral defenses among Native Americans. In contemporary settings, exposure to novel pathogens (*e.g*., SARS-CoV-2) and interactions with nuclear variants from diverse ancestries (“new genetic signatures”), whether additive or epistatic, may further modulate risk, such that a neutral ancestry in its original genomic background can yield unanticipated outcomes in a new one. 

Furthermore, in line with this model, Nunes et al. (2025) reported >8.5 million novel variants, including >36,000 predicted deleterious alleles, in their Brazilian cohort, many enriched on Indigenous local-ancestry genomic regions. This previously underrepresented variation expands the space for interaction effects, increasing the potential for mismatch between ancestral adaptations and contemporary environments. Conversely, as detailed elsewhere in this review, evidence of post-contact natural selection has been observed, including in Indigenous-ancestry segments (Nunes *et al*., 2025).

There are cases in which “witness genetic signatures” are detectable only in admixed populations after depopulation or extermination, with admixed groups acting as reservoirs of Indigenous lineages (Marrero et al., 2007; Tavares et al., 2019). In southern Brazil, mitochondrial DNA analyses of *Gaúchos* from the Pampas of the state of Rio Grande do Sul (which borders Argentina and Uruguay) showed that ~52% of lineages are of Indigenous origin, an unusually high proportion for Brazil, second only to Amazonia (Marrero *et al*., 2007). Within this Native American component, the prominence of haplogroup C is consistent with signals of *Charrúan* ancestry. The *Charrúa*, an Indigenous group from the Southern Cone that historically occupied the Pampas of present-day Uruguay and neighboring regions of Brazil and Argentina, have long been considered extinct since the nineteenth century; yet their maternal lineages persist among modern *Gaúchos* as “witness genetic signatures” indicating genetic continuity in the Pampas despite severe demographic collapse (Marrero *et al*., 2007). Moreover, mitogenomes from a *Gaúcho* sample outside the Pampas, assigned to the Native American haplogroup C1d3 (Tavares et al., 2025), are being investigated as potential remnants of *Charrúa* ancestry, a finding that underscores the enduring genetic legacy of this historically diminished yet remarkable people of the Pampas and exemplifies very well the notion of “witness genetic signature.”

Extending this reasoning beyond mtDNA, nuclear genome-wide data can likewise retain “witness signatures” of Indigenous history. Using local ancestry inference in more than 5,800 individuals from three Brazilian cities (Salvador, Bambuí, and Pelotas), we virtually reconstructed chromosomes entirely of Native American ancestry (Mas-Sandoval et al., 2019). The reconstructed genomes separated according to the deep split between Tupi- and Jê-speaking peoples, consistent with linguistic and archaeological records. Tupi-related ancestry was concentrated along the Brazilian coast, reflecting historical eastward expansions, whereas Jê-related ancestry predominated in the interior, indicative of their long-standing presence in central Brazil. However, our analyses also indicated that the pre-colonial eastern coast of Brazil was not a continuous Tupi territory. Instead, the reconstructed genomes from the Salvador sample showed a relatively strong Jê signal, whereas those from the southern coast (Pelotas) displayed greater Tupi-Guarani affinity. These patterns reveal both contact and discontinuities between Jê and Tupi groups along the coast before 1500, challenging the notion of a continuous Tupi corridor. Importantly, this approach enabled the recovery of the histories of Native American peoples that no longer exist as distinct communities, including coastal populations severely affected or collapsed during the European colonization (Mas-Sandoval *et al*., 2019). Thus, “witness genetic ancestry” present in admixed individuals, with their preserved fragments of both Tupi and Jê ancestries, offer a unique opportunity to reconstruct the trajectories of Indigenous peoples whose histories would otherwise have been lost (Mas-Sandoval *et al*., 2019).

In the case of “transplanted genetic signatures”, the pattern is exemplified by low-admixture migrant communities whose genetic profiles remain largely preserved. For instance, in Veranópolis, a small city strongly shaped by nineteenth-century Italian immigration, both maternal and paternal markers are almost exclusively European (Marrero et al., 2005), reflecting limited integration into the broader admixture process that shaped most Brazilian populations.

By contrast, the high frequency of European mtDNA and Y chromosomes in admixed Brazilians (Carvalho-Silva et al., 2001; Nunes et al., 2025) represents “introduced genetic signatures”, formed through extensive gene flow during the early colonial period. African components incorporated through the transatlantic slave trade likewise form part of these “introduced signatures”, with African ancestry detectable in both uniparental systems (Hünemeier et al., 2007; Gonçalves et al., 2008).

A regionally distinctive example concerns the Y-chromosome profile of *Gaúchos*, which shows striking affinities with Spanish rather than Portuguese lineages, unlike most of Brazil, consistent with the colonial and geopolitical history of Rio Grande do Sul, where control alternated between Spanish and Portuguese empires (Marrero et al., 2007). In our Y-SNP/STR study, *Gaúcho* paternal heritage more closely resembles that of Spaniards, fitting the expected pattern of “introduced signatures” shaped by local historical contingencies.

Figure 2 also highlights nuclear examples consistent with our framework. First, the 32-bp CCR5-Δ32 deletion, which reduces HIV-1 entry, is a “introduced genetic signature” whose frequency closely tracks European ancestry across Brazilian admixed populations (Ellwanger et al., 2020; Kulmann-Leal et al., 2021). Ellwanger *et al*. (2020) also review CCR5-Δ32 and broader CCR5 modulation across viral infections beyond HIV. One origin estimate (~682 years before present) overlaps the Black Death in Europe (1347-1352) and has been cited to argue for a medieval selective event capable of elevating CCR5-Δ32 from rarity to ~10% in present-day Europeans (O’Brien, 2024). However, recent ancient-DNA work places the origin much earlier, roughly 8,000-2,000 years ago in western Eurasia, undermining both the Black Death and Viking-dispersal hypotheses while remaining consistent with long-term positive selection (Ravn et al., 2025). Second, the lactase persistence allele (13910 C>T in an enhancer within the neighboring gene *MCM6* that regulates *LCT*, the lactase gene) shows the same pattern, that is, it was introduced into Brazil through European ancestry (Mattar et al., 2009). Notably, lactase persistence arose convergently in pastoralist populations, where adult consumption of milk and dairy products conferred an adaptive advantage; multiple regulatory variants emerged, including in East Africa, but in admixed Brazilians the putative European 13910T predominates (Mattar *et al*., 2009). Similarly, *APOL1* risk variants G1 and G2 represent African “introduced genetic signatures”: selected in West/Central Africa for protection against trypanosomes that, in homozygotes or compound heterozygotes (G1/G1, G2/G2, or G1/G2), are associated with increased risk of kidney disease. These variants reached Brazil via the transatlantic slave trade (Daneshpajouhnejad et al., 2022; Giudicelli et al., 2022). Notably, all “introduced genetic signatures” carry evolutionary histories forged on other continents.

We can also cite more recent examples of “witness” and “introduced genetic signatures” identified by Nunes et al. (2025). The same study highlights the context of “new genetic signature” (mosaic genomes), where clear signals of post-admixture selection are detected, *i.e*., although genomic segments derive from different continental ancestries, the adaptive sweeps occurred in America after the admixture events. “Witness genetic signatures” include candidate nuclear genes, such as *ACSS1* and *MTMR3* (immune pathways) and *USP3* (DNA repair). “Introduced signatures” of European origin include *LINC00871* (fertility), *AATF*/*ACACA* (menopause), *ZNF430* (transcriptional regulation), and *LILRs* (immunity). “Introduced signatures” of African show similar patterns, exemplified by *ZNF184*/*POM121L2* (spermatogenesis) and *ARHGAP11A* (sleep duration). Collectively, these results illustrate how unique demographic events and local selective pressures have sculpted the mosaic genomes of contemporary admixed populations in the Americas.

## Genetic adaptations of Native Americans to the American environment

As the first human groups entered America, a continent of striking environmental diversity, natural selection acted on settlers encountering novel habitats and shaping adaptive traits. Many were marked by extreme temperatures and humidity, intensely cold or hot, excessively wet or arid, alongside environmental hypoxia and high ultraviolet radiation (UVR) exposure, placing multiple pressures on the earliest Indigenous populations. On the other hand, temporal shifts in lifestyle and diet, from ancient foraging and the agricultural ecologies of the Andean highlands and Mesoamerica to modern regimes, may even influence susceptibility to contemporary diseases. Perspectives on these and related themes appear in Salzano (2016) and Hünemeier et al. (2025). As noted, “witness genetic signatures” capture not only demographic history but also clear signatures of natural selection, as discussed below.

### Adapting to new diets: metabolic challenges in Native American populations

Colonizing new environments imposed metabolic challenges, especially via dietary shifts. A prominent example is *ABCA1* Arg230Cys (rs9282541), a derived allele found exclusively in Native Americans and their descendants, associated with increased risk of diabetes and obesity. The 230Cys variant reduces cholesterol efflux by ~27% *in vitro*, correlates with lower HDL and higher BMI, and shows strong signatures of positive selection, initially framed as a “thrifty” adaptation conferring energetic or infection-related advantages under scarcity and early sedentary/incipient urban settings (Acuña-Alonzo et al., 2010). Its frequency tracks the archaeology of maize domestication in Mesoamerica, peaking in long-established farming groups such as the Zapotec and Maya, consistent with early, less diversified agriculture and recurrent famine in maize-dependent sedentary societies. This represents one of the earliest well-documented cases of gene-culture coevolution in America: maize-based subsistence favored energy-storage variants that today increase susceptibility to metabolic disease (Hünemeier et al., 2012).

Parallel adaptive signals, potential markers of adaptive epistasis, are evident in tropical-forest people. In the Suruí and Karitiana, Amorim et al. (2015) detected positive selection on *SCP2* and *CWH43*, genes involved in lipid transport and metabolism, consistent with adaptation to nutritional instability in rainforest settings. These results indicate that metabolic adjustments also shaped hunter-gatherer societies living in pathogen-rich, resource-variable ecosystems.

At the continental scale, selection has acted on the *FADS* gene cluster, which encodes enzymes critical for polyunsaturated-fatty-acid metabolism. In Inuit, a selected *FADS* haplotype is associated with adaptation to marine, lipid-rich diets (Fumagalli et al., 2015). Separately, adaptive introgression from Denisovans was identified at *TBX15/WARS2*, implicated in cold adaptation and body-fat distribution (Racimo et al., 2017). In our study (Amorim et al., 2017), the selected *FADS* haplotype is nearly fixed across Native American populations, including the ancient Anzick-1 individual (~12.5 kya), supporting a selective sweep likely during the Beringian standstill rather than a signal restricted to Inuit contexts. Originally advantageous under glacial conditions and lipid-rich diets, this variant persists in contemporary Native American groups living in diverse ecologies, illustrating the long-lasting imprint of ancient nutritional challenges. The widespread *FADS* signal across America is consistent with first settlers carrying and dispersing the adaptive haplotype as they colonized varied environments; the forces maintaining or amplifying it beyond Arctic/Beringian settings were likely distinct, reflecting a “witness genetic signature” shaped by shifting diets and ecological/pathogen landscapes.

### Immunologic and pathogen-driven adaptations in the new environment

The settlement of the American continent exposed Native American populations to a novel pathogenic landscape, requiring immunological adaptations to cope with unfamiliar infectious agents and conditions encountered in these new environments. Early immunogenetic studies already pointed to adaptive dynamics. Veit et al. (2012) showed balancing selection at the *HLA-G* 14-bp InDel polymorphism in South American groups, particularly among Macro-Tupi speakers. The heterozygote excess observed likely reflects the dual role of *HLA-G* in fetal survival and immune regulation, suggesting pathogen-mediated balancing selection in reproductive and immunological contexts. Genome-wide analyses further reinforced the role of immune adaptation. Amorim et al. (2015) identified signals of positive selection in Suruí and Karitiana, including *CCL28* and immune pathways such as PD-1 and IL-12 signaling, consistent with the high pathogen burden of rainforest environments.

A unique temporal perspective was provided by Lindo et al. (2016), who sequenced exomes from a Northwest Coast population before and after European contact. They showed that *HLA-DQA1* alleles were nearly fixed in ancient individuals but declined sharply in modern descendants, suggesting that variants advantageous against endemic pathogens became maladaptive in the face of European-borne diseases. This study exemplifies how colonial epidemics reshaped Native American immunogenetic adaptations.

Broader *HLA* variation has also been characterized. Single et al. (2020) reported endemic alleles and signatures of long-term balancing selection in *HLA-A, -B, -C,* and *-DRB1* across the American continent. While diversity maintenance is consistent with pathogen-driven balancing, the marked regional differentiation indicates local adaptation to specific microbial environments. In addition, Ojeda-Granados et al. (2022) demonstrated polygenic adaptation in Mexican Indigenous groups, with networks involving metabolism, immunity, and pathogen response, including *Trypanosoma cruzi* and *Leishmania mexicana*.

Other studies highlight pleiotropic and functional mechanisms. Mendoza-Revilla et al. (2022) found selection in immune loci such as *HLA-DQA1, OAS1,* and *TLR1*, reinforcing the role of both viral and bacterial exposures. Finally, Couto-Silva et al. (2023) provided functional evidence of adaptation in *PPP3CA*, showing that reduced expression decreases *T. cruzi* infectivity in cardiomyocytes, a variant whose distribution correlates with reduced Chagas disease incidence in the Amazon.

Studying cytokines, key modulators of immune responses, Zembrzuski et al. (2010) found that among the Xavante Indigenous people, despite high BCG vaccine coverage, most individuals showed tuberculin skin test anergy, suggesting that host genetic factors may increase susceptibility to tuberculosis caused by members of the *Mycobacterium tuberculosis* complex (MTBC). Ancient DNA from pre-Columbian Peru indicates a pre-contact presence of MTBC, with the most recent common ancestor dating to roughly 6 kya; however, these ancient strains are no longer found in present-day human populations and were likely replaced by the *M. tuberculosis* lineage introduced by European colonists into America (Orgeur et al., 2024). Together, these findings represent yet another set of “witness genetic signatures”, documenting a long adaptive trajectory within the continent. 

### Living in the heights

The colonization of high-altitude regions by Andeans represents a hallmark of human adaptation to extreme environments. Although these regions were among the last landscapes to sustain permanent human settlement (Salzano, 2019), archaeological and genetic evidence indicates initial human presence in the Andes as early as ~12 kya (Jolie et al., 2011; Rademaker et al., 2014; Fehren-Schmitz et al., 2017). Above 2,500 meters, reduced barometric pressure causes hypobaric hypoxia, while intense ultraviolet radiation (UVR) and large diurnal thermal amplitudes impose severe physiological stress (Moore, 2001; Rademaker *et al*., 2014). As emphasized by Julian and Moore (2019), Andean highlanders display distinctive physiological profiles, including enlarged lung volumes, a reduced hypoxic ventilatory response, and increased uterine blood flow during pregnancy, and, under acute exposure, show early contraction of plasma volume followed by slower increases in total red blood cell volume and total hemoglobin mass; total blood volume changes little, but hemoglobin concentration (and thus oxygen-carrying capacity) rises. Studies of Indigenous groups such as the Aymara, Quechua, and Colla document these multiple adaptations, with elevated hemoglobin concentration as a principal hypoxia-related response (Stuber and Scherrer, 2010; Beall, 2007; Bigham and Lee, 2014; Valverde et al., 2015; Eichstaedt et al., 2015), and this response scales with altitude: red blood cell volume is ~17-23% higher at 3,600-3,800 m and ~50-60% higher at 4,300-4,500 m than in lowlanders (Champigneulle et al., 2024 and references therein).

Compared with other highlanders, Andeans rely most on hematological adjustment, with high hemoglobin (≈18-20 g/dL), high hematocrit (~54%), and a relatively low hypoxic ventilatory response. Tibetans have a contrasting strategy, lower hemoglobin (≈14-16 g/dL), higher lung volumes, stronger ventilatory response, slightly larger red cells, and elevated nitric oxide, with modestly lower oxygen saturation. Ethiopian highlanders appear intermediate in hemoglobin (≈15-16 g/dL) and generally show low ventilation, though several metrics are not reported. Overall, Andeans emphasize blood-based compensation, Tibetans ventilatory and vascular adjustments, and Ethiopians moderate profiles relative to the others (Bigham et al., 2010; Roche et al., 2024; Seifu et al., 2025).

These and other high-altitude pressures are reflected in the genome. In Andeans, selection signals cluster on genes involved in oxygen sensing and vascular/hematologic regulation, including *EGLN1* (also implicated in Tibetans), *VEGFA* and *PDGFRB* (angiogenesis/vascular growth), and *CYP17A1* (steroidogenic control of vascular tone). Notably, *EGLN1* encodes an enzyme that degrades HIF-α and thereby tunes erythropoiesis and ventilation. Positive selection also affects *EPAS1*, where the missense variant rs570553380 (His→Arg) appears Andean-specific (Lawrence et al., 2024); its relatively young age and modest frequency suggest early-stage selection in Andean highlanders. By contrast, many Tibetan groups carry *EPAS1* variants at high frequency or near fixation, consistent with an older, stronger selection episode; the adaptive Tibetan *EPAS1* haplotype derives from Denisovan introgression (>48 kya) and reflects a longer high-altitude occupation (Lawrence *et al*., 2024), while in Ethiopian highlanders the genetic pathways involved (Seifu et al., 2025) are consistent with their intermediate physiological profiles relative to the others.

Overall, the evidence points to convergent solutions alongside distinct, population-specific routes to the same selective pressures, culminating in genetic signatures that are sometimes similar and sometimes divergent. A notable finding from our group is the implication of proximal and extended *TP53* pathway genes in Andeans. *TP53* encodes the p53 transcription factor, which safeguards genome integrity by regulating programs for cell-cycle control, DNA repair, apoptosis, senescence, and metabolism; when *TP53* or its pathway is disrupted, cancer risk rises (mutations occur in >50% of human tumors). We hypothesize that in harsh high-altitude environments, marked by chronic hypoxia, intense UVR, and large diurnal temperature swings, genes within this network may be co-opted to support long-term human adaptation.

In a first study, using a candidate-gene strategy, we analyzed five polymorphisms in the *TP53* pathway (*TP53*, *MDM2*, *MDM4*, *USP7*, *LIF*; Jacovas et al., 2015) in 282 Andean and lowland Native Americans and incorporated published data from 100 additional individuals. We found altitude- and UVR-associated shifts in allele frequencies (notably *USP7*-G and *LIF*-T, and, in the expanded set, *MDM2*-T) and an enrichment of the *MDM2*-TT genotype at high altitude, consistent with positive selection on *TP53*-network genes for Andean high-altitude adaptation. *MDM2*-TT homozygotes express typical steady-state levels of MDM2, maintain adequate p53, and can appropriately respond to environmental stresses (Jacovas *et al*., 2015). Moreover, because hypoxia stabilizes p53 through MDM2 down-regulation, these results further support *MDM2*-TT as an adaptive genotype at high altitude. Multilocus interaction analyses also revealed a central role for *LIF* and *USP7*, indicating that high-altitude adaptation is better explained by synergistic interactions among alleles of different genes (adaptive epistasis) rather than by *TP53* alone. 

In a second study, we expanded from a candidate-gene approach to a genome-wide selection scan (analyzing approximately 214,000 SNPs) comparing long-term Andean highlanders with Amazonian and Mesoamerican lowlanders, and identified three new loci under positive selection in Andeans, *SP100*, *DUOX2*, and *CLC,* which are genes of the extended *TP53* pathway (Jacovas et al., 2018). Signals from multiple tests of natural selection based on population differentiation and haplotype structure coincided with altitude-graded allele frequencies, and functional follow-ups indicated increased expression of *SP100* (notably in skeletal muscle) and *DUOX2* (especially in lung and arterial tissues), consistent with roles in genomic stability, oxidative-stress handling, thyroid hormone biology, and angiogenesis under chronic hypoxia. Together with prior findings on the *TP53* network, these results indicate that Andean high-altitude adaptation resulted from coordinated shifts across multiple pathways rather than from isolated routes, displaying distinctive patterns such as the evolutionary co-option of genes not observed in other highland groups.

*SP100* emerged as an intriguing surprise. One SNP, rs13411586, is associated with increased SP100 protein production, suggesting an evolutionary tuning that helps balance cellular responses to hypoxia and mitigate the harms of prolonged low oxygen, potentially optimizing muscle function and angiogenesis (Jacovas et al., 2018). The *SP100* gene encodes a nuclear protein that interacts with p53, modulating its activity. Genomic analyses indicate that the C allele of rs13411586 is under positive selection and reaches high frequency in high-altitude Andean populations (Jacovas *et al*., 2018). *In silico* predictions further suggest that this variant alters SP100 expression patterns, conferring advantages in hypoxic environments (Jacovas *et al*., 2018). Beyond hypoxia, *SP100* has also been implicated in cardiovascular adaptation: studies in ischemic cardiomyopathy models link reduced *SP100* expression to mitochondrial dysfunction and heart failure, pointing to a critical interplay with the HIF-1 pathway (Herrer et al., 2015). These findings position *SP100* as a central “witness genetic signature” in adaptation to high-altitude stressors and offer a fresh lens on the molecular mechanisms underlying human evolution in extreme environments.

However, functional studies are essential to validate the role of genetic variants in cellular responses to hypoxia and to determine whether, and how, they influence physiological and adaptive processes. CRISPR/Cas9-based approaches have enabled the functional interrogation of variants showing signals of positive selection in human populations (Xu et al., 2014). In a research line recently developed by our group, we integrate evolutionary analyses with molecular characterization of adaptive mechanisms, focusing on the *SP100-*C allele highlighted by Jacovas et al. (2018), which showed the strongest signal of selection in the Andeans. 

**Figure 3:**
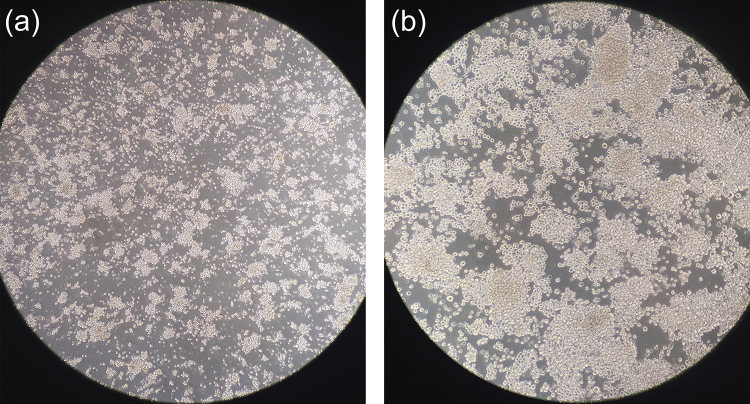
K562 cells exposed to hypoxia (5% O₂) for 10 days. (a) Wild-type cells showing smaller clusters and moderate confluence; (b) cells edited to carry the *SP100*-C allele (knock-in) showing larger clusters and high confluence. These results are preliminary and should be interpreted with caution; independent replication and additional cell models are needed and are being conducted.

Using gene editing through CRISPR/Cas9 and additional K562 cell-line models with specific treatments (Table S1), we observed that *SP100-*C increases cell viability under simulated hypoxia (5% O₂) *in vitro* and confers greater proliferative capacity than cells carrying the allele predominant in lowland populations (Figure 3). Under normoxia (21% O₂; day 14), both lineages expanded, with higher total counts in the Andean lineage (~3,883,333) than in the wild type (~2,423,333), while viability remained high (cell death 13-36%). Under hypoxia, the Andean lineage again achieved a larger total count (~1,340,000 *vs*. ~906,333 in wild type) but exhibited higher mortality (mean 54.67% *vs*. 34.33%). By day 10, edited cells showed better morphology and greater confluence; however, extending hypoxia to day 14 likely pushed cultures past a growth limit (density-driven stress/medium acidification), obscuring the earlier advantage. These preliminary findings suggest a difference in growth between cell lines carrying the TT and CC genotypes in context of hypobaric hypoxia and support the hypothesis that high-altitude selective pressures can have shaped a distinct genetic profile in Andean populations. Independent replications and additional cell models are underway to confirm or refute these results. The Supplementary Material and Methods present the details of the methods used to generate the preliminary results presented here.

The role of the *TP53* network in human adaptation has been increasingly recognized, with evidence spanning from cellular to population levels and involving reproduction, longevity, and environmental stress responses. This growing body of evidence reinforces our findings in Andean populations and aligns with recent insights highlighting the multilevel adaptive functions of *TP53* (Voskarides and Giannopoulou, 2023).

In another study from our team (Jacovas et al., 2022), we investigated the genetic diversity of the *HLA-G* 3′UTR in 17 South American Indigenous populations, comparing high-altitude Andean communities with lowland groups from the Amazon, Chaco, and the Brazilian Central Plateau to understand adaptation to hypoxia. HLA-G is a non-classical MHC class I molecule that functions as an immune checkpoint, promoting immune tolerance and being responsive to hypoxia, with expression documented both in physiological (maternal-fetal interface) and pathological (tumoral) hypoxic microenvironments. Ten haplotypes were identified, most notably UTR-2 and UTR-5, whose frequencies correlated with altitude: UTR-2 was more frequent in Andean populations (47%) and positively associated with altitude, suggesting advantage under chronic hypoxia; conversely, the ancestral UTR-5 predominated in lowland groups (21.5%) and was negatively associated with altitude, consistent with adaptation to tropical environments with high pathogen loads. Neutrality tests indicated a trend toward balancing selection, supporting the long-term maintenance of functional variants that modulate soluble HLA-G levels in response to environmental pressures.

These findings represent an evolutionary legacy of a “witness signature” with current public-health implications: the same genetic repertoire that supports hypoxia tolerance and reproductive success of Andean populations may, in long-lived modern populations, increase susceptibility to cancer under pathological hypoxia by facilitating tumor immune escape. Indeed, this has been suggested for Andean populations in Peru indicate that the rate of gestational and postpartum complications in Aymara Andean regions is lower than the national average, while the incidence of cancer in highland populations, including the Andeans, is higher (Jacovas et al., 2022, and references therein). 

Our hypothesis (Jacovas et al., 2022) aligns with the concept of late-acting antagonistic pleiotropy, the idea that the same genetic repertoire can enhance early-life fitness yet impose late-life costs. Our Native American case study is consistent with prior findings and illustrates this mechanism particularly well, showing how variants favored for hypoxia tolerance and reproductive success may contribute to disease vulnerability later in life. As summarized by Austad and Hoffman (2018), these trade-offs are common, perhaps ubiquitous, a view supported by accumulating genetic evidence, now including our findings in Native American populations.

### Skin pigmentation and vitamin D-folate hypothesis

Human skin pigmentation reflects a polygenic adaptation whose evolutionary trajectories were largely independent across continents, yielding mostly distinct sets of associated alleles in Africa, Europe, and East Asia (Liu et al., 2024). Compared to other continental populations, the history of skin color adaptation among Native Americans is less well known (Missaggia et al., 2020). Although a few pigmentation alleles are shared across regions, such as the *MFSD12* missense variant Tyr182His, which is common in both East Asians and Native Americans and associated with lighter pigmentation (Adhikari et al., 2019), ancient DNA evidence indicates that most skin-lightening processes in East Asians took place after their divergence from Native Americans, suggesting that pigmentation evolved along distinct trajectories in the two groups after their separation (Ferrando-Bernal, 2023). Consequently, the allele architecture influencing pigmentation in Native Americans is likely, at least in part, to be distinct from that of other regions and remains less well characterized: in the Kalinago, Native American genetic ancestry is associated with skin-color differences, yet the underlying variants did not map to previously catalogued pigmentation loci (Ang et al., 2023). Targeted work is beginning to fill these gaps, for instance, signals consistent with positive selection at *MITF*, a key regulator of melanocyte development and pigmentation, have been reported in Andean highlanders (Caro-Consuegra et al., 2022). To situate these genetic observations, it is useful to review the ecological logic of UVR-mediated selection on pigmentation.

Ultraviolet radiation is widely recognized as the principal selective pressure shaping human skin pigmentation, as this trait correlates more strongly with UVR intensity than with any other environmental variable (Chaplin, 2004). Because UVR levels are typically higher at low latitudes and high altitudes, Indigenous populations inhabiting such environments generally exhibit darker skin than those living elsewhere (Jablonski and Chaplin, 2010). However, Native Americans often deviate from these global patterns. For instance, Indigenous peoples of the Arctic display darker pigmentation than would be predicted based on their latitude, whereas high-altitude Andeans tend to be lighter-skinned than expected for their geographic setting (Missaggia et al., 2020). These departures make Native American skin color an especially compelling subject for further investigation.

Some authors attribute the mismatch between Native American skin color and local UVR to the relatively recent settlement of America, arguing there has been insufficient time for full adaptation (Jablonski, 2004; Juzeniene et al., 2009; Quillen, 2015; Adhikari et al., 2019; Rocha, 2020). By contrast, the vitamin D-folate hypothesis posits that selection on pigmentation is mediated by UVR’s physiological effects: UVR-B promotes cutaneous vitamin D synthesis, whereas UVR-A accelerates folate photodegradation. Because melanin is photoprotective, higher eumelanin would be adaptive under intense UVR to limit folate loss, while reduced eumelanin would be favored under low UVR to facilitate vitamin D production. This framework further implies that genes involved in vitamin D and folate metabolism also contribute to adaptation to local radiation environments.

Given this context, Native American pigmentation patterns may partly reflect adaptation via pathways beyond canonical pigmentation loci. Building on this, a prior study from our group (Missaggia et al., 2020) used variant-level network analysis to link pigmentation genes with components of the vitamin D and folate metabolism pathways, reporting putative co-adaptive signatures that differed between agriculturalists (principally Andean) and hunter-gatherer populations. Although preliminary, these results motivate cross-pathway analyses of UVR-related adaptation. To deepen this line of inquiry, an ongoing investigation within our team is applying interpretable machine-learning methods to compare the relative importance of pigmentation, vitamin D, and folate metabolism pathways in Native American and other continental autochthonous populations. Initial findings are consistent with the hypothesis advanced by Missaggia *et al*. (2020), indicate distinct adaptive responses to ultraviolet radiation between the population sets and suggest alternative evolutionary solutions to similar environmental pressures. As this research is still in progress, the findings remain preliminary, but they highlight a promising avenue for understanding the complexity of local adaptation in America.

## Future perspectives: metagenomics and microbiomes

The current era has witnessed a rapid expansion of omics-based studies. Genomic and metagenomic research can underpin a more effective and inclusive precision medicine, and Indigenous peoples must not be excluded from these advances. The oral microbiota offers an additional lens to understand Indigenous health disparities as we have previously highlighted (Marcano-Ruiz et al., 2023). The human oral ecosystem has co-evolved with its host through complex dynamics in which eubiosis can shift to dysbiosis, contributing to periodontal disease and caries. Periodontal diseases are a major global public health concern: since 1990, incidence, prevalence, and burden (measured in disability-adjusted life years), have risen by nearly 8%, with the greatest impact in countries with low human development indices (Cui et al., 2022). Dysbiosis is also linked to systemic conditions of high social and public health relevance, including cardiovascular and metabolic disorders such as type 2 diabetes, and though still debated, has been associated with neurodegenerative diseases, including Alzheimer’s disease (Marcano-Ruiz *et al*., 2023).

A study of an isolated Indigenous group, the Yanomami, by Clemente et al. (2015) showed that antimicrobial-resistance genes can be detected in the absence of clinical antibiotic exposure. Yet Indigenous peoples remain severely underrepresented in microbiome research, perpetuating inequities in preventive care and in the development of precision-health interventions. Consistent evidence shows that untreated dental caries, periodontitis, and limited access to restorative services disproportionately affect Indigenous groups, underscoring the need to integrate microbiome and genomic research into culturally sensitive health strategies.

Considering these aspects, there is a clear need to include Native populations in metagenomic research. For example, Arantes et al. (2021) documented temporal shifts in oral-health patterns in the Kadiwéu: adults exhibited lower caries indices, whereas children and adolescents showed worse indicators, suggesting a recent decline likely related to increasing contact with urban culture. In this context, studying the oral microbiota of Indigenous populations is essential both to reduce inequities and to inform medical technologies designed for these communities, as well as to evaluate the impact of modern diets on the oral ecosystem. 

Building on this rationale, our group, together with collaborating teams, is undertaking a collective effort to generate metagenomic profiles across distinct Amazonian hunter-gatherer groups to help fill a critical knowledge gap with clear implications for public health and, ideally, to contextualize microbiome dynamics and their resistomes amid recent changes in lifestyle and diet, drawing on longitudinal notes and reports initiated by Professor Francisco Mauro Salzano; these datasets are currently being produced.

## Supplementary material

The following online material is available for this article:

Table S1 - Experimental design of the study. Wild-type (WT) and edited (EDT) cell populations were cultured under standard oxygen levels (N) and hypoxic conditions (H) for 0 or 14 days.

Figure S1 - Major population flows that shaped the American continent from pre-Columbian times to the early 20th century.

Supplementary Material - for the “Living in the Heights” section.

## Data availability

 No original genetic data were used in this work.
