# Supplementary material for: Genetic insights into the peoples who shaped the American continent
Source: Genet Mol Biol. 2026 Apr 3;49(Suppl 1):e20250244. doi: 10.1590/1678-4685-GMB-2025-0244 (PMC13063108; doi:10.1590/1678-4685-GMB-2025-0244)
Supplement: Table S1 - [file 1415-4757-GMB-49-s1-e20250244-s1.pdf]

Genetics and Molecular Biology

https://doi.org/10.1590/1678-4685-GMB-2025-0244

Supplementary Material to "Genetic insights into the peoples who shaped the American continent"

Table S1 - Experimental design of the study. Wild-type (WT) and edited (EDT) cell populations were cultured under standard oxygen levels (N) and hypoxic conditions (H) for 0 or 14 days.

Designation	Cell type	Oxygen level	Time in cell culture
WT-N-0	WT	Standard	0 days
EDT-N-0	Edited	Standard	0 days
WT-N-14	WT	Standard	14 days
EDT-N-14	Edited	Standard	14 days
WT-H-14	WT	Hypoxia	14 days
EDT-H-14	Edited	Hypoxia	14 days

Note: Standard (21% O_2); Hypoxia (5% O_2). Although atmospheric O_2 remains ~21% at all altitudes, reduced barometric pressure in the Andes lowers inspired O_2, from ~20.9% at sea level to ~13–14% at 3,500–4,000 m (*e.g.*, Cusco, La Paz). *In vitro*, 5% O_2 approximates physioxia (~1–6%), providing a biologically relevant minimum for viable cellular metabolism.
