# Supplementary material for: Genetic insights into the peoples who shaped the American continent
Source: Genet Mol Biol. 2026 Apr 3;49(Suppl 1):e20250244. doi: 10.1590/1678-4685-GMB-2025-0244 (PMC13063108; doi:10.1590/1678-4685-GMB-2025-0244)
Supplement: Figure S1 - [file 1415-4757-GMB-49-s1-e20250244-s2.pdf]

Genetics and Molecular Biology

https://doi.org/10.1590/1678-4685-GMB-2025-0244

Supplementary Material to "Genetic insights into the peoples who shaped the American continent"

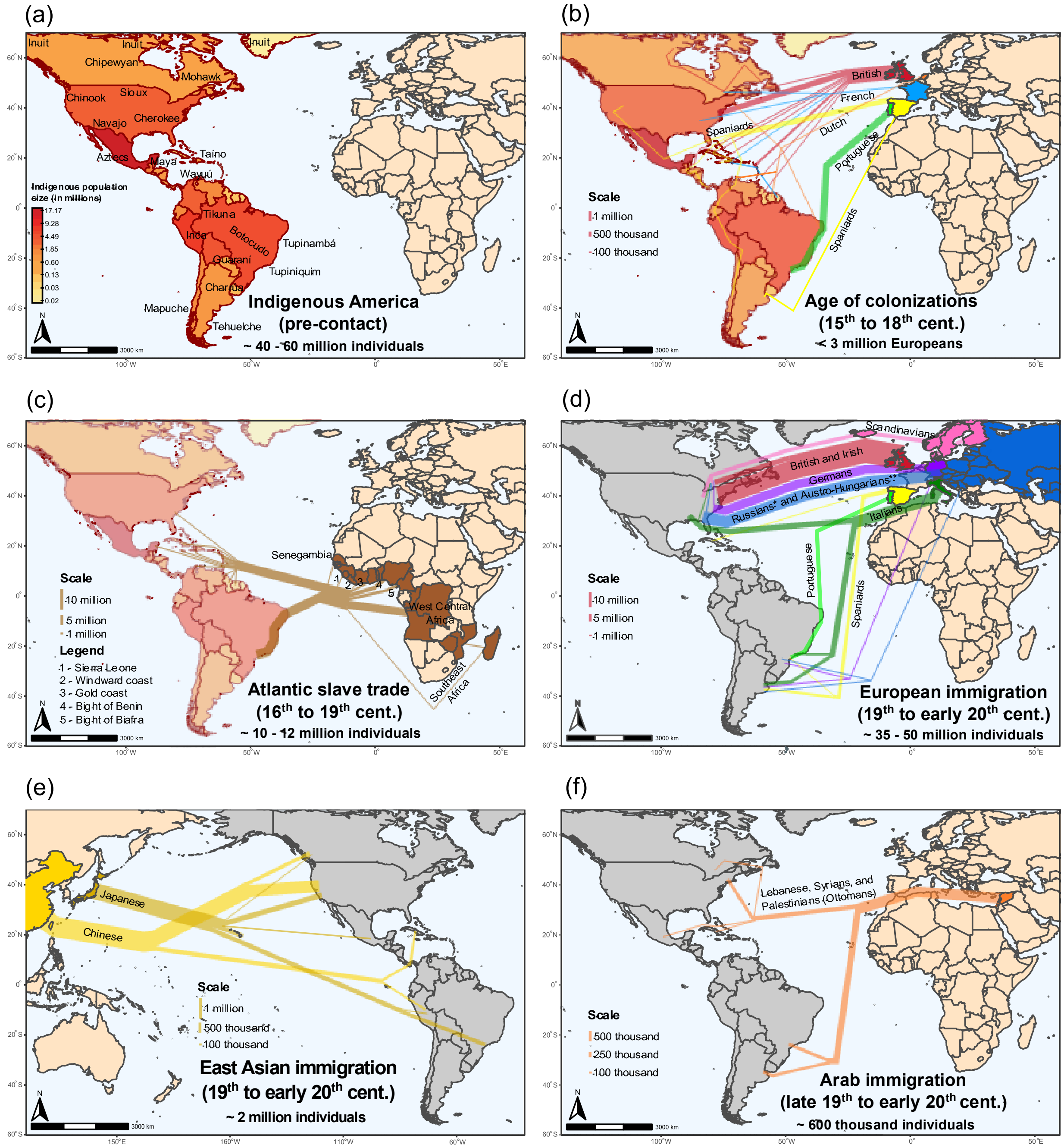

Figure S1 – Major population flows that shaped the American continent from pre-Columbian times to the early twentieth century. Panels summarize approximate timing, origins, destinations, and demographic magnitudes of successive migratory waves: (a) Indigenous America before European contact (~40–60 million individuals), with selected well-known Indigenous groups indicated; (b) European colonization during the Age of Discoveries (15th–18th centuries; <3 million Europeans); (c) the Atlantic slave trade (16th–19th centuries; ~10–12 million enslaved Africans); (d) mass European immigration (19th to early 20th centuries; ~35–50 million), primarily from the British Isles, Italy, Germany, the Austro-Hungarian Empire (including its subject countries), the Russian Empire (notably Poles and Jews), Spain, Portugal, and Scandinavia; (e) East Asian immigration (19th–early 20th centuries; ~2 million Chinese and Japanese); and (f) Arab immigration (late 19th–early 20th centuries; ~600,000 Lebanese, Syrians, and Palestinians under Ottoman rule). Arrows schematize broad zones of origin and principal directions of movement rather than precise routes. Panel-specific numeric and spatial scales may differ to enhance readability. Numbers and scale reflect broad demographic estimates based on historical and demographic sources (Curtin, 1972; Blanchard, 1979; Klein, 1983; Daniels, 1986; Nugent, 1992; Hu-DeHart, 1993; Kunimoto, 1993; Rosoli, 1994; Salzano and Bortolini, 2002; Alonso, 2007; Instituto Brasileiro de Geografia e Estatística, 2007; Moya and McKeown, 2010; Hooper and Batalova, 2015; Adhikari et al., 2017; Fahrenthold, 2019; Fortes-Lima and Verdu, 2021; Botelho, 2025; Library of Congress, 2025; Lyman Museum and Mission House, 2025; Nunes et al., 2025). Contemporary political boundaries are shown for orientation only. Additionally, smaller flows are not depicted; for example, Chile and Uruguay were also primary receivers, though in smaller proportions than the destinations highlighted.

References

Adhikari K, Chacón-Duque JC, Mendoza-Revilla J, Fuentes-Guajardo M and Ruiz-Linares A (2017) The genetic diversity of the Americas. Annu Rev Genomics Hum Genet 18:277-296.

Alonso BS (2007) The other Europeans: Immigration into Latin America and the international labour market (1870–1930). Rev Hist Econ 25:395-426.

Blanchard P (1979) Asian immigrants in Peru, 1899–1923. North-South Can J Lat Am Stud 4:60-75.

Botelho T (2025) População e imigração no Brasil, 1808-1920. Hist Econ Hist Empresas 28:165-201.

Curtin PD (1972) The Atlantic slave trade: A Census. University of Wisconsin Press, Madison, 357 p.

Daniels R (1986) Chinese and Japanese in North America: The Canadian and American experiences compared. Can Rev Am Stud 17:173-188.

Fahrenthold SD (2019) Arab labor migration in the Americas, 1880–1930. In: Dailey J (ed) Oxford Research Encyclopedia of American History, Oxford Academic, New York, pp 1-22.

Fortes-Lima C and Verdu P (2021) Anthropological genetics perspectives on the transatlantic slave trade. Hum Mol Genet 30:R79-R87.

Hooper K and Batalova J (2015) Chinese immigrants in the United States, Migration Policy Institute, https://www.migrationpolicy.org/article/chinese-immigrants-united-states-2013 (accessed 20 September 2025).

Hu-Dehart E (1993) Chinese coolie labour in Cuba in the nineteenth century: Free labour or neo-slavery? Slavery Abol 14:67-86.

IBGE - Instituto Brasileiro de Geografia e Estatística (2007) Brasil: 500 anos de povoamento. IBGE, Rio de Janeiro, 232 p.

Klein HS (1983) The integration of Italian immigrants into the United States and Argentina: A comparative analysis. Am Hist Rev 88:306-329.

Kunimoto I (1993) Japanese migration to Latin America. In: Stallings B and Székely G (eds) Japan, the United States, and Latin America. Palgrave Macmillan, London, pp 99–121.

Library of Congress (2025) Immigration and Relocation in U.S. History, https://www.loc.gov/classroom-materials/immigration/ (accessed 20 September 2025).

Lyman Museum & Mission House (2025) Women's History Month: Japanese Women in Hawai'i, https://lymanmuseum.org/exhibits/archive-special-exhibits/womens-history-month-japanese-women-in-hawai%CA%BBi/ (accessed 20 September 2025).

Moya JC, McKeown A (2010) World migration in the long twentieth century. In: Adas M (ed) Essays on Twentieth-Century History. Temple University Press, pp 9-52.

Nugent WT (1992) Crossings: The Great Transatlantic Migrations, 1870–1914. Indiana University Press, Bloomington, 364 p.

Nunes K, Araújo Castro e Silva M, Rodrigues MR, Lemes RB, Pezo-Valderrama P, Kimura L, Schenatto de Sena L, Krieger JE, Varela MC, de Azevedo LO et al. (2025) Admixture's impact on Brazilian population evolution and health. Science 388:eadl3564.

Rosoli, G. (1994). 14: The Global Picture of the Italian Diaspora to the Americas. Center for Migration Studies Special Issues 11:305-322.

Salzano FM and Bortolini MC (2002) The evolution and genetics of Latin American populations. Cambridge University Press, Cambridge, 530 p.
