# Supplementary material for: Genetic insights into the peoples who shaped the American continent
Source: Genet Mol Biol. 2026 Apr 3;49(Suppl 1):e20250244. doi: 10.1590/1678-4685-GMB-2025-0244 (PMC13063108; doi:10.1590/1678-4685-GMB-2025-0244)
Supplement: Supplementary Material - [file 1415-4757-GMB-49-s1-e20250244-s3.pdf]

Genetics and Molecular Biology

https://doi.org/10.1590/1678-4685-GMB-2025-0244

Supplementary Material to "Genetic insights into the peoples who shaped the American continent"

Material and Methods for the "Living in the Heights" section

CRISPR/Cas9 and gRNA design

The SpyFi CRISPR/Cas9 Nuclease system (Aldevron) (Lot: MPM021-08) was used for genetic editing to generate a cell population with predefined genotypes: the high-frequency genotype in the Andean population (CC) for the *SP100* SNP rs13411586. As the wild-type (WT) cell population, we used the most frequent genotype in the general population (TT). In this system, the Cas9 nuclease, a guide RNA (gRNA) homologous to the target region, and a DNA template fragment containing the variant to be inserted are included. Genome editing was performed using the gRNA sequence 5'TCTGAAGTGCCATCCCCTCTGG 3' and a donor DNA template containing the sequence: 5' ATTCAGTAGAATGCCATTTGGAAAACACCGTTCTGAA GCGCCATCCCCTCTGG CCCCTGAATCCTCACCAGC TTCCA 3'.

The gRNA design was performed using the CRISPOR tool (http://crispor.gi.ucsc.edu/), which enables the selection of specific sequences for genomic editing. gRNAs near the PAM (NGG) motif were prioritized, with high cutting efficiency and a low risk of off-target effects, based on genomic specificity and editing efficiency analyses. The tool also evaluates homology with other genomic regions, ensuring that the selected gRNA has high precision and efficiency in editing, minimizing the possibility of undesired modifications.

Cell line and culture

The cell line used for the editing experiment was K562, an immortalized lymphoblastoid cell line derived from a patient with chronic myeloid leukemia (CML) in the blast crisis phase. These non-adherent cells are easy to culture and are characterized by aneuploidy, with variable chromosomal content. Under optimal conditions, K562 cells exhibit rapid growth, with a doubling time of approximately 24 hours, making them convenient for studies requiring a large number of cells in a short period (Lozzio and Lozzio, 1975). For culture and expansion, the cells were maintained in T25 culture flasks with RPMI medium supplemented with 10% fetal bovine serum and 1% penicillin/streptomycin at 37°C in a 5% CO_2 atmosphere.

Transfection and vectors

Aliquots of 250,000 cells were separated for nucleofection using the electroporation technique. This process uses electrical pulses to form temporary pores in the cell membrane, facilitating the entry of plasmids, ribonucleoprotein complex (RNP), and DNA template directly into the nucleus with high efficiency (Gresch *et al*., 2004). For the first group of cells, plasmids carrying only GFP were delivered, while another group simultaneously received Cas9 and guided RNA assembled into a ribonucleoprotein (RNP) complex, along with a DNA template for gene editing with homology-directed repair (HDR). The plasmid vector containing the gene encoding the green fluorescent protein (GFP) was used to observe the success of the nucleofection technique, analyzing both the reagents used and the program set for the cell line on the Lonza 4D-Nucleofector system. For the RNP complex, a molar ratio of 1:2.4 (Cas9 + gRNA, respectively) was used. Both samples were incubated in a culture incubator with 5% CO_2 at 37°C in Recovery Medium (RPMI, 20% fetal serum, 2% penicillin/streptomycin, and 2% Glutamax). The nucleofected cells with RNP were treated for 48 hours with the reagent AZD7648 (1uM) at, which acts as a selective inhibitor of DNA-dependent protein kinase (DNA-PK), blocking DNA double-strand break repair by non-homologous end joining (NHEJ) and facilitating knock-in editing (HDR) (Selvaraj *et al*., 2024).

Clonal population selection by flow cytometry and molecular analysis of clones

The cells transfected with the plasmid were analyzed under a fluorescence microscope for three days post-transfection, confirming the presence of cells expressing GFP, indicating the success of the nucleofection process. For the cells treated with RNP, the editing efficiency was evaluated by sequencing PCR-amplified fragments, with the sequences analyzed using the ICE-SYNTHEGO software (https://ice.synthego.com). This software provides a simple and accurate analysis to determine the variety and frequency of targeted mutations generated in a cell population, utilizing raw sequencing files with electropherograms obtained from Sanger sequencing on the ABI3500 instrument. Its algorithm reconstructs the indel profile from sequence data, and the web interface displays the identity and frequencies of the detected indels.

Subsequently, the nucleofected cells with RNP underwent single-cell sorting by flow cytometry using the BD FACSMelody equipment. The cells were seeded individually into 96-well plates with culture medium supplemented with 10% fetal bovine serum and 2% penicillin/streptomycin and incubated at 37°C with 5% CO2 to allow clonal growth. Once approximately 40% confluence was reached, the cells were transferred to larger 24-well plates, and when they reached 100% confluence, about 50% of the cells were collected for DNA extraction. The DNA was amplified by PCR using primers Forward: 5’AGAGAACAAGAAGCGACCGAG 3’ and Reverse: 3’TAGAAACAGTGACCTTCGCCC 5’, and the samples were purified according to the laboratory protocol and sent for Sanger sequencing (ABI3500 genetic analyzer and BigDye Terminator v3.1 reaction, both from ThermoFisher Scientific). The resulting sequences were analyzed and compared to verify the editing for each population. Clones with scores close to 100% knock-in for the target allele were selected and expanded.

Functional pilot study

The functional characterization of both genotypes was preliminarily evaluated in stages by means of live cell and total cell counts for the WT and edited cell populations subjected to different experimental conditions (normoxia- 21% O_2; and hypoxia-5% O_2) in a 14-day pilot study. A total of 500,000 cells were seeded in six-well plates, forming triplicates for the six experimental groups, as shown in Table S1. Throughout the 14-day experiment, culture medium changes were performed as needed, with centrifugation of the medium at 1,200 rpm for 5 minutes to collect non-adherent cells suspended in the medium, which were then returned to the wells after the medium change. All culture plates were handled simultaneously to ensure consistency across experimental conditions. Cell counting on the fourteenth day was performed using a Neubauer chamber with the Countess 3 automatic cell counter. Images of the hypoxia-treated cells were recorded on the tenth day.

Noteworthy, although the atmospheric oxygen fraction remains approximately 21% at all altitudes, the markedly lower barometric pressure in the Andes reduces the partial pressure of inspired O_2, effectively decreasing physiological O_2 availability—from an equivalent ~20.9% at sea level to ~13–14% at elevations of ~3,500–4,000 m (*e.g.*, Cusco and La Paz). *In vitro*, cultures were maintained under 5% O_2, as most mammalian tissues function under physioxic conditions (~1–6% O_2), whereas standard incubator conditions (18–21% O_2) correspond to supraphysiological hyperoxia. The 5% O_2 setting thus provides a biologically relevant approximation of *in vivo* O_2 tension while maintaining cell viability (Stuart *et al.*, 2018).

References

Gresch O, Engel FB, Nesic D, Tran TT, England HM, Hickman ES, Körner I, Gan L, Chen S, Castro-Obregon S et al. (2004) New non-viral method for gene transfer into primary cells. Methods 33:151-163.

Lozzio CB and Lozzio BB (1975) Human chronic myelogenous leukemia cell-line with positive Philadelphia chromosome. Blood 45:321-334.

Selvaraj S, Feist WN, Viel S, Vaidyanathan S, Dudek AM, Gastou M, Rockwood SJ, Ekman FK, Oseghale AR, Xu L et al. (2024) High-efficiency transgene integration by homology-directed repair in human primary cells using DNA-PKcs inhibition. Nat Biotechnol 42:731-744.

Stuart JA, Fonseca J, Moradi F, Cunningham C, Seliman B, Worsfold CR, Dolan S, Abando J and Maddalena LA (2018) How supraphysiological oxygen levels in standard cell culture affect oxygen-consuming reactions. Oxid Med Cell Longev 2018:8238459.
